# Lung cancer screening an asbestos exposed population: Existing lung cancer risk criteria are not sufficient

**DOI:** 10.1111/resp.14487

**Published:** 2023-03-08

**Authors:** Fraser J. H. Brims, Edward J. A. Harris, Conor Murray, Chellan Kumarasamy, Alice Ho, Brendan Adler, Peter Franklin, Nick H. de Klerk

**Affiliations:** ^1^ Department of Respiratory Medicine Sir Charles Gairdner Hospital Perth Western Australia Australia; ^2^ Curtin Medical School Curtin University Perth Western Australia Australia; ^3^ National Centre for Asbestos Related Diseases Institute for Respiratory Health Perth Western Australia Australia; ^4^ ChestRad Medical Imaging Perth Western Australia Australia; ^5^ Envision Medical Imaging Perth Western Australia Australia; ^6^ School of Global and Population Health University of Western Australia Perth Western Australia Australia

**Keywords:** asbestos exposure, LDCT screening, lung cancer, lung neoplasms, occupational exposure

## Abstract

**Background and Objective:**

Asbestos is a major risk factor for lung cancer, with or without tobacco smoke exposure. Low dose computed tomography (LDCT) screening for early lung cancer is effective but only when targeting high risk populations. This study aimed to analyse the effectiveness of LDCT screening in an asbestos exposed population and to compare lung cancer screening program (LCSP) eligibility criteria.

**Methods:**

Participants in an asbestos health surveillance program, the Western Australia Asbestos Review Program, underwent at least one LDCT scan and lung function assessment as part of annual review between 2012 and 2017. Lung cancer cases were confirmed through linkage to the WA cancer registry. Theoretical eligibility for different screening programs was calculated.

**Results:**

Five thousand seven hundred and two LDCT scans were performed on 1743 individuals. The median age was 69.8 years, 1481 (85.0%) were male and 1147 (65.8%) were ever‐smokers (median pack‐year exposure of 20.0). Overall, 26 lung cancers were detected (1.5% of the population; 3.5 cases per 1000 person‐years of observation). Lung cancer was early stage in 86.4% and four (15.4%) cases were never smokers. Based on current lung screening program criteria, 1299 (74.5%) of this population, including the majority (17, 65.4%) of lung cancer cases, would not have been eligible for any LCSP.

**Conclusion:**

This population is at raised risk despite modest tobacco exposure. LDCT screening is effective at identifying early‐stage lung cancer in this population and existing lung cancer risk criteria do not capture this population adequately.

## Graphical abstract

This prospective cohort study of 1743 asbestos exposed individuals demonstrates that the population is at high risk for lung cancer regardless of smoking status, low dose CT screening is effective at identifying early‐stage lung cancer in this population and existing lung cancer risk criteria do not capture this population adequately.

**See related** Editorial

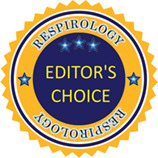

## INTRODUCTION

Lung cancer is the second most common cancer in the world (11.4% of all cancers) but the leading cause of cancer death (18.0% of total cancer deaths).^1^ In Australia, lung cancer is the 5th most commonly diagnosed cancer, but remains the highest cause of cancer death and has the highest burden associated with any cancer.^2^

In addition to age and tobacco smoke exposure,^3^ lung cancer risk is associated with factors such as environmental and genetic interactions, second hand smoke, biofuel, indoor and outdoor pollution and occupational exposures.^4^, ^5^, ^6^ Asbestos was widely used in most developed countries in the last century and Australia had some of the highest global rates of asbestos utilization and exportation.^7^ Asbestos is an important occupational cause of lung cancer with asbestos exposure known to increase the risk for lung cancer even in people who have never smoked.^8^ In those with both asbestos and tobacco smoke exposure, the increased risk is more than additive and up to multiplicative.^8^ The population attributable risk for asbestos contributing to lung cancer is estimated to be up to 30%.^4^, ^9^, ^10^

The detection of early‐stage lung cancer using low dose computed tomography (LDCT) is effective in reducing deaths from lung cancer, but only in selected high‐risk populations.^11^, ^12^ Adoption of formal lung cancer screening program (LCSP) has been slow internationally, with the practicalities of implementation, health economics and optimal eligibility criteria remaining concerns for many countries.^13^

Eligibility for lung cancer screening can be defined by fixed criteria such as the United States Preventative Services Taskforce (USPSTF) that utilize age 50–80 years, a tobacco exposure of more than 20 pack‐years, and less than 15 years since quitting.^14^ The Liverpool Lung Project (LLP) risk model accounts for additional risk factors such as personal history of cancer, family history of lung cancer and reported asbestos exposure.^15^, ^16^ The Prostate Lung Colon Ovarian lung cancer risk model (PLCO_m2012_) is a similar risk‐based model derived from a large north American population that utilizes multiple variables to derive a 6‐year risk of developing lung cancer, with the optimal PLCO risk for a LCSP thought to be >1.51%.^17^ This has been prospectively evaluated in the International Lung Screening Trial.^18^ In Australia, two different eligibility criteria for a LCSP have been proposed. Cancer Australia has proposed an age bracket of 55–74 (with lower age reduced to >50 years for indigenous people) and a PLCO_m2012_ 6 year lung cancer risk of >1.51%,^13^ while the Medical Services Advisory Committee (MSAC) has proposed an age bracket of 55–70, a tobacco exposure of more than 30 pack‐years, and less than 10 years since quitting.^19^ The recently proposed UK program will include eligibility from the PLCO_m2012_ or LLP models^20^ (see Table 1 for a summary).

**TABLE 1:** Eligibility for lung cancer screening programs.

Name	Criteria	Abbreviated criteria
USPSTF_2013_	Aged 55–80, ≥30 pack year history and <15 years since quitting	55–80–30–15
USPSTF_2021_	Aged 50–80, ≥20 pack year history and <15 years since quitting	50–80–20–10
PLCO_m2012_	6‐year lung cancer risk (utilizing variables: age, ethnicity, tobacco exposure, educational level, BMI, COPD, personal cancer history and family history of lung cancer) aged 50–80	50–80 > 1.51
CA LCSP	Aged 55–74, PLCO_2012_ 6‐year lung cancer risk >1.51%	55–74 > 1.51
MSAC LCSP	Aged 55–70, ≥30 pack year history and <10 years since quitting	55–70–30–10
UK LCSP	PLCO_2012_ risk >1.51% or LLPv.3 risk ≥2.5%	55–74 > 1.51 > 2.5

Abbreviations: CA, Cancer Australia; LCSP, lung cancer screening program; MSAC, Medical Services Advisory Committee; PLCO, Prostate Lung Colon Ovarian; UK, United Kingdom; USPSTF, United States Preventative Services Taskforce.

The Western Australia Asbestos Review Program (ARP) has been studying asbestos‐exposed individuals since 1990 with chest x‐ray (CXR) as part of the annual assessment. Since inception, 4240 individuals have attended the ARP on at least one occasion. In 2012, LDCT was adopted in preference to CXR as part of the annual review.^21^ The aim of this study was to analyse the effectiveness of the first 5 years of the LDCT program and to examine LCSP eligibility criteria against the ARP population.

## METHODS

### Study design

The ARP is an annual health surveillance program for individuals who have had significant occupational and environmental exposure to asbestos. It has been described in detail previously.^22^, ^23^ Originally, the cohort was established to monitor the health of crocidolite miners and workers, and local township residents. Other participants are required to have a ≥3 months cumulative asbestos exposure history and/or the presence of pleural plaques (or other asbestos related disease) on imaging to be eligible (a mixed‐occupation, mixed‐asbestos fibre exposed cohort). This cohort is drawn from predominantly primary care and self‐referrals to the program.

The ARP annual health assessment includes health and lifestyle questionnaires, lung function (spirometry and DLCO) and LDCT imaging. A spirometry diagnosis of chronic obstructive pulmonary disease (COPD) was accepted with a predicted FEV_1_ < 80% and FEV_1_/FVC ratio <0.7. Tobacco smoking history, including duration and pack‐year exposure, is updated at each annual visit. Individual exposures to asbestos have been estimated (Appendix S1 in the Supporting Information).

### Subjects and follow‐up period

All participants must have had at least one LDCT scan and lung function assessment, usually performed within 1–2 days of each other. This report analyses 5 years of LDCT screening from August 2012, with a subsequent 1 year of follow‐up nodule surveillance (surveillance year), through to August 2018.

### Protocol for LDCT screening scans

All CT scans were performed prone at 1.5 mm slice thickness using a Siemens Definition (2012–2013), Siemens Definition Flash (2013–2014) or Siemens Force (2014—2017) machine (Siemens Healthineers, Munich, Germany). The technical and reporting protocol is described in detail in Appendix S1 in the Supporting Information.

### Nodules and lung cancer

Indeterminate nodules were defined as solid, part‐solid or non‐solid nodules without benign‐type calcification greater than a mean dimension of 5 mm. The Computer Aided Detection software was utilized as a routine adjunct to clinical reporting. Follow‐up of nodules was decided by the clinical team. The PanCan nodule malignancy risk model^24^ was applied retrospectively to the nodules as part of this report.

A diagnosis of lung cancer was accepted with a histological or cytological report, a clinical diagnosis by multidisciplinary (MDT) consensus, and/or from record data linkage (see below). Staging was in accordance with the TNM Classification for Lung Cancer 8th edition.^25^

### Eligibility assessment

Theoretical eligibility for different screening programs was calculated (but not used for recruitment: see Table 1).

### Case ascertainment

Cancer incidence and type was obtained from the Western Australian Cancer Registry, using ICD‐10 code C34.9 for lung cancer. Mortality was assessed by linking the cohort to the Western Australian Registrar General for births, deaths and marriages. The censor date for survival was 01 September 2018.

### Statistical analysis

Continuous variables were assessed for normality and their distribution expressed in terms of mean and standard deviation if parametric or median and interquartile range if non‐parametric. Categorical variables were described in terms of number and percentage of total. Comparisons of continuous variables was made with the Mann–Whitney *U* test, *t*‐test and one way ANOVA; the Chi‐Square test was used to compare proportions. The proportions of participants eligible for different LCSPs was calculated with the numbers needed to screen and person‐years of follow‐up to detect one lung cancer. Never smokers were not included in the PLCO_m2012_ calculation; the LLP calculation assumed all family history of lung cancer cases occurred >60 years (conservative). Data was analysed using IBM SPSS Statistics v.26 (SPSS Inc., Chicago, IL).

## RESULTS

Between 2012 and 2017, 5702 LDCT scans were performed on 1743 individuals. The median age of participants was 69.8 years (IQR 63.0–75.7), 1481 (85.0%) were male and 1147 (65.8%) were ever‐smokers. Table 2 summarizes the demographics of the cohort, stratified by exposure category. Participants' region of origin was recorded as Australasia for 1121 (64.3%) and Europe for 565 (32.4%). One participant was aboriginal. Overall, there was 7417 person‐years of follow‐up. CXR was performed in the year prior to LDCT in 939 (53.9%). For all participants, median (IQR) PLCO_m2012_ lung cancer risk was 0.71 (0.25–2.08)% and LLP 2.0 (0.90–4.53)%. Over the 5‐year screening period, 84 participants (4.8%) of the cohort withdrew from the program, the vast majority citing difficulty in travelling to the program through increasing age.

**TABLE 2:** Demographic characteristics of study population, stratified by asbestos exposure.

	All cohort	Mixed‐fibre exposure	Ex‐residents	Wittenoom workers	*p*‐value
*n* =	1743	1155	363	225	N/A
Median age at T0 LDCT, years (IQR)	69.8 (63.0–75.7)	69.9 (64.0–76.2)	62.7 (55.0–70.8)	74.5 (71.1–78.1)	<0.005
Male *n* = (%)	1481 (85.0)	1095 (94.8)	180 (49.6)	206 (91.6)	<0.005
Ever smokers *n* = (%)	1147 (65.8)	773 (66.9)	200 (55.1)	174 (77.3)	<0.005
Median (IQR) pack years—ever smokers	20.0 (8.4–37.5)	17.5 (8.0–33.0)	21.9 (7.2–40.3)	32.0 (16.4–58.6)	<0.005
TSFE, years (SD)	51.3 (9.1)	51.1 (10.4)	50.8 (7.5)	52.9 (4.1)	0.02
Median cumulative asbestos exposure‐fibre/mL years (IQR)	0.7 (0.1–3.0)	0.3 (0.01–1.0)	3.2 (1.3–7.6)	6.1 (1.9–19.5)	<0.005
Presence of pleural plaque at T0 *n* = (%)	1066 (61.1)	779 (67.5)	131 (36.1)	156 (69.3)	<0.005
Presence of asbestosis at T0 *n* = (%)	602 (34.5)	451 (39.1)	55 (15.1)	96 (42.7)	<0.005
FEV_1_% predicted	92.4 (80.1–104.0)	92.5 (80.2–104.0)	94.0 (82.7–105.1)	89.0 (71.8–102.4)	0.338
FVC% predicted	95.0 (83.4–106.8)	93.5 (82.6–104.4)	102.9 (89.2–111.1)	89.9 (79.4–105.2)	0.239
FEV_1_/FVC ratio	0.74 (0.68–0.78.6)	0.74 (0.68–0.78)	0.76 (0.71–0.80)	0.72 (0.65–0.78)	0.188
DLCO % predicted	88.9 (73.4–103.1)	88.9 (74.3–104.2)	92.1 (78.0–103.4)	75.3 (62.3–83.4)	0.524
Lung cancer	26 (1.5)	15 (1.3)	6 (1.7)	5 (2.2)	0.556

Abbreviations: DLCO, gas transfer; FEV_1_, forced expiratory volume in 1 second; FVC, forced vital capacity; IQR, interquartile range; LDCT, low dose computed tomography; T0, baseline scan; TSFE, time since first (asbestos) exposure.

Asbestosis was present on CT in 602 (34.5%) of the population and 1066 (61.1%) had pleural plaques, confirming significant asbestos exposure. Emphysema was present on CT in 254 (14.5%) individuals and spirometry‐defined COPD present in 206 (11.8%) of the population. The median DLP of annual (screening) scans over the study period was 14 mGy cm (range 10–36) which is an effective dose of 0.2 mSv.

Overall, 26 lung cancers were identified over the study period (1.5% of the total population). The rate of detection of lung cancer was 3.5 cases per 1000 person‐years of observation. Nineteen lung cancers were detected on LDCT within the five‐year CT period. A further three cases had nodules detected at year 5 with lung cancer diagnosed during the following surveillance year. Data linkage identified four further lung cancers that were diagnosed in individuals who had withdrawn from the ARP. Of these, three were interval cancers (not visible on the most recent ARP LDCT), and one had a 4 mm nodule on the last LDCT, in retrospect. The characteristics of all 26 lung cancers are presented in Table 3, with comparison to the remainder of the cohort.

**TABLE 3:** Summary of demographics for ARP cohort, stratified by lung cancer diagnosis.

	Lung cancer (*n* = 26), *n* = (% or IQR)	No lung cancer (*n* = 1717), *n* = (% or IQR)	*N* =	*p* =
*Basic demographics*				
LDCT detected lung cancer	22	‐	1743	
Lung cancer diagnosed outside screening	4	‐		
Male sex	23 (88.5)	1458 (84.9)	1743	0.61
Age at first scan (years)	75.4 (68.4–80.8)	69.7 (62.8–75.7)	1743	<0.005
Age at cancer diagnosis scan (years)	77.2 (71.3–81.5)	‐	26	
First degree relative—lung cancer	5 (27.8)	15 (0.9)	1603	<0.005
PLCO_m2012_ 6‐year risk %	2.24 (0.80–4.32)	0.68% (0.25–2.00)	933	0.082
LLP risk %	3.40 (1.70–11.0)	1.90 (0.90–4.40)	1500	0.105
*Tobacco exposure at LDCT enrolment*				
Ex	18 (69.2)	1010 (58.8)	1028	0.049
Current	4 (15.4)	115 (6.7)	119	
Never	4 (15.4)	592 (34.5)	596	
Tobacco exposure (pack‐years)	25.5 (17.4–54.7)	20.0 (8.4–37.3)	1743	0.037
*Cohort group*				
Mixed fibre (mixed occupations)	15 (57.7)	1140 (66.4)	1155	0.56
Ex‐Wittenoom worker	5 (19.2)	220 (12.8)	225	
Ex‐Wittenoom resident	6 (23.1)	357 (20.8)	363	
*Asbestos exposure history*				
Time since first exposure (years)	50.2 (45.6–53.2)	51.7 (46.7–57.5)	1402	0.37
Median cumulative asbestos exposure‐fibre/mL years (IQR)	1.37 (0.12–2.10)	0.72 (0.09–2.98)	1677	0.49
*CT findings at baseline*				
Radiological asbestosis	11 (42.3)	591 (34.4)	602	0.40
Pleural plaques	18 (69.2)	1048 (61.1)	1066	0.39
Diffuse pleural thickening	4 (15.4)	152 (8.9)	156	0.24
Emphysema visible	10 (38.5)	244 (14.2)	254	0.001
*Lung function at baseline*				
FEV_1_% predicted	85.6 (77.2–105.0)	92.4 (80.1–104.0)	1704	0.338
FVC% predicted	90.1 (75.9–105.2)	95.0 (83.5–106.9)	1703	0.239
FEV_1_/FVC ratio	0.71 (0.67–0.77)	0.74 (0.68–0.78.6)	1703	0.188
DLCO % predicted	77.7 (61.7–88.7)	88.9 (73.4–103.1)	1321	0.524
*Histology*				
Adenocarcinoma	14 (53.8%)	‐		
Squamous	5 (19.2%)	‐		
Adenosquamous	3 (11.5%)	‐		
Carcinoid	1 (3.8%)	‐		
NSCLC not otherwise specified	1 (3.8%)	‐		
Clinical	2 (7.7%)	‐		

Abbreviations: DLCO, gas transfer; FEV_1_, forced expiratory volume in 1 second; FVC, forced vital capacity; IQR, interquartile range; LDCT, low dose computed tomography; LLP, Liverpool Lung Project lung cancer risk model; NSCLC, non‐small cell lung cancer; PLCO_m2012_, Prostate Lung Colon Ovarian lung cancer risk model.

Of the 22 cancers detected on LDCT, 17 were detected at baseline (first LDCT; prevalent) and five were identified during the follow‐up period. Nineteen (86.4%) of the cancers were stage IA or IB, one was stage IIA and two stage IVA. Surgery (video assisted thoracoscopic lobectomy) with curative intent was performed in 13, four had stereotactic radiotherapy, three participants had chemoradiation and two best supportive care. All lesions operated on were malignant. One participant died 4 months after surgery (found to have M1a disease during operation). Mutation analysis was performed on five tumours: two were positive for KRAS mutation (both in never smokers), one PIK3CA, one TP53, one wildtype.

### Pulmonary nodules

A total of 199 indeterminate nodules were detected in 187 individuals, see Table 4. Over the full follow‐up period, 127 of the nodules were stable, 49 had resolved or reduced in size, 22 were diagnosed as lung cancer, and 1 oligometastatic melanoma.

**TABLE 4:** Demographic features of participants with and without lung nodules.

	Nodule(s) present	No nodules
	*n* = (%), or median (IQR)	
Number of individuals	187 (10.7)	1556 (89.3)
Number of pulmonary nodules	199	‐
Age at detection scan (years)	71.6 (65.6–77.1)	‐
Prevalent (detected on T0 scan)	148 (74.3)	‐
Incident	51 (25.6)	‐
*Tobacco exposure*		
Ex	114 (61.0)	534 (34.3)
Current	12 (6.4)	107 (6.9)
Never	61 (32.6)	534 (34.3)
*Tobacco smoke exposure in smokers (pack years)*	19.5 (7.6–45.0)	17.0 (7.0–33.0)
*Asbestos exposure history*		
Mixed fibre (mixed occupations)	107 (57.2)	1048 (67.4)
Ex‐Wittenoom worker	35 (18.7)	190 (12.2)
Ex‐Wittenoom resident	45 (24.0)	318 (20.4)
*CT findings*		
Asbestosis	63 (33.7)	539 (34.6)
Pleural plaques	119 (63.6)	947 (60.9)
Diffuse pleural thickening	18 (9.6)	138 (8.9)
Emphysema	33 (17.6)	221 (14.2)
*Nodule characteristics*		
Mean nodule diameter (mm)	6 (Range 5–10)	‐
Non‐solid	35 (17.6)	‐
Part‐solid	34 (17.1)	‐
Solid	130 (65.3)	‐
Spiculated	11 (5.5)	‐
Upper lobe location	79 (39.7)	‐
PanCan risk of nodule at detection (%)	1.7 (0.6–6.8)	‐
*Other risk factors*		
First degree relative—lung cancer	1 (0.5)	14 (0.8)
Patient reported COPD	21 (11.2)	111 (7.1)
Spirometry‐defined COPD	67 (35.8)	558 (35.9)
*Planned nodule management*		
6 week follow‐up CT	4 (2.0)	‐
3 month follow‐up CT	115 (57.8)	‐
6 month follow‐up CT	14 (7.0)	‐
PET scan	12 (6.0)	‐
Contrast CT	5 (2.5)	‐
Continue annual screening	49 (24.6)	‐

Abbreviations: COPD, chronic obstructive pulmonary disease; IQR, interquartile range; LDCT, low dose CT scan; PanCan Risk, probability of a nodule being diagnosed as a lung cancer in next 2–4 years; PET, positron emission tomography; T0, baseline scan.

### Eligibility for other screening programs

Post‐hoc analysis of eligibility and numbers of lung cancers detected dependent on different screening program and risk criteria are presented in Table 5 (the breakdown of variables are presented in Table S1 in the Supporting Information). One thousand two hundred ninety‐nine (74.5%) of the population would not have been eligible for any LCSP based mainly on pack‐years of smoking. The majority (17, 65.4%) of lung cancers would not have been detected by any eligibility criteria.

**TABLE 5:** Numbers of participants meeting eligibility, numbers of lung cancers detected and numbers needed to screen to detect one lung cancer stratified by different lung cancer screening criteria.

Criteria	*N* = individuals meeting criteria for screening (*n* = 1743)	*N* = lung cancers detected (*n* = 26)	Number needed to screen to detect 1 lung cancer	Person years of follow‐up to detect 1 lung cancer
USPSTF_2013_ (55–74–30–15)	164 (9.4%)	5 (19.2%)	33	128
USPSTF_2021_ (50–80–20–10)	200 (11.5%)	7 (26.9%)	29	113
PLCO_m2012_ (50–80 > 1.51)	262 (15.0%)	8 (30.8%)	33	133
CA LCSP (55–74 > 1.51)	183 (10.5%)	4 (15.4%)	46	190
MSAC LCSP (55–70–30–10)	76 (4.4%)	2 (7.7%)	38	150
LLP (55–74 ≥ 2.5)	388 (22.3%)	6 (23.1%)	65	272
UK proposed LCSP (55–74 > 1.51 > 2.5)	444 (25.5%)	9 (34.6%)	49	208

Abbreviations: CA, Cancer Australia; LCSP, lung cancer screening program; MSAC, Medical Services Advisory Committee; PLCO, Prostate Lung Colon Ovarian lung cancer risk model; USPSTF, United States Preventative Services Taskforce; UK, United Kingdom (utilizes either/or PLCO and LLP models).

### Survival

Consistent with the aging nature of the population there were 121 (6.9% of total enrolled) deaths during the 6‐year study period. Of the 26 participants with lung cancer, 10 had died before the date of censor, with a median (IQR) survival for all lung cancer cases of 720 (343–1797) days; for cases who had surgery, the median survival was 1558 (583–1842) days.

## DISCUSSION

This study demonstrates that LDCT screening of an asbestos‐exposed population can identify early‐stage lung cancer in individuals who otherwise may not have been eligible for lung cancer screening. In this population over half had been screened with CXR in the year prior (including 50% of the subsequent LC cases), nearly one third were never smokers and of the ever‐smokers the tobacco exposure is mostly below eligibility for existing LCSPs. This relatively modest tobacco exposure likely accounts for the comparatively lower lung cancer cases per person‐years compared to other screening studies.^11^, ^26^ A significant majority of this population (74.5%) would have been ineligible for any current or proposed LCSP, including 65.4% of the lung cancers cases. This highlights the importance of the risk conferred by asbestos exposure, independent of tobacco smoke exposure.

Other studies examining outcomes in asbestos exposed populations have reported lung cancer incidence between 1.2% and 2.6%,^27^, ^28^, ^29^ with differing risk from different asbestos fibre types well recognized.^30^ A recent analysis of the entire ARP population (since inception) demonstrated that the presence of asbestosis confers an increased adjusted risk for lung cancer, but presence of pleural plaque does not.^31^ Asbestos is accounted for in some risk models,^15^, ^32^, ^33^ although none of these account for the additive synergism of combined tobacco and asbestos exposure.

The strength of this cohort study is how well phenotypically described the population is, and the comprehensive, data‐linked follow‐up. The participants with lung cancer had a greater tobacco smoke exposure, a higher estimated cumulative asbestos exposure and more emphysema visible on CT, as compared to those without lung cancer. The PLCO_m2012_ and LLP model risk appeared higher but the difference did not reach statistical significance (Table 3).

The USPSTF recently broadened their eligibility criteria to specifically include a *lower* risk population after simulation‐based modelling demonstrated that LDCT screening could lead to important reductions in lung cancer mortality and significant life years gained if the US population was optimally targeted.^14^, ^34^ In our analysis, the USPSTF_2021_ had the lowest numbers needed to screen and person years of follow‐up to detect one lung cancer, however the optimal eligibility model was the proposed UK model (utilizing PLCO and/or LLP scores), with 26% of the ARP population eligible and 35% of lung cancers detected. This is likely to be driven by the LLP model including an asbestos exposure variable to inform risk. The least effective criteria to identify lung cancers in the present study's population is the Australian MSAC LCSP, detecting just 8% of the cancers (and 4% of the ARP population).

Further research to address the knowledge‐implementation gap to enable a practical, feasible approach to account for occupational asbestos exposure in targeted lung cancer screening programs is needed. A time‐based estimate of cumulative asbestos exposure could be a pragmatic approach for LCSPs to assess for eligibility,^35^ with the ARP eligibility criteria being more than 3 months cumulative full‐time exposure to asbestos.

The ARP population is comprised of just under two thirds of a mixed fibre, mixed occupation (mostly industrial) asbestos cohort, and while the impact of crocidolite exposure from the Wittenoom mine and township remains evident, these ex‐miners and residents are not over‐represented in the detected lung cancers. As such, the results of this study are likely generalizable to wider asbestos exposed populations, with previous estimates of at least 410,000 Australians occupationally exposed to asbestos.^10^

In this cohort, indeterminate pulmonary nodules were found in 10.7% of participants over the CT screening period. This is lower than other lung cancer screening studies, presumably related to the definition of a nodule, relatively low tobacco exposure in the cohort and perhaps geography.^12^, ^18^ All screen‐detected nodules in this study were <10 mm, presumably with larger nodules previously detected by CXR or as a recruitment bias with no nodules of concern on CT prior to entry into the ARP. The vast majority of the nodules were either stable or resolved over the follow‐up period, with one quarter continuing annual screening, and 57.8% having 3‐month interval imaging. It should be noted that at inception of the program guidance such as LungRads^36^ and the PanCan malignancy risk model^24^ were not published and so the clinicians adopted what is now likely to be an overcautious, conservative approach on the outset. The surveillance of pulmonary nodules is a highly significant component in the total cost of a potential screening program and the use of a clear guideline based approach is critical to ensure safe and efficient follow‐up.^37^ Overall, the ARP protocol and approach would suggest this is an effective program, with good participant retention, relatively low numbers of nodules (with a high number nodules being malignant [11.1%]) and most (86.4%) cancers being early stage. A formal cost‐effectiveness evaluation is required.

As expected for a screening population, this study identified a predominance of adenocarcinomas, which raises the likelihood of overdiagnosis.^38^ The ARP was not designed to be a lung cancer screening program and has always been a health surveillance program, hence there is no upper age limit for participants. This study has not attempted to examine any independent risk factors associated with lung cancer or the presence of nodules with multivariate logistic regression or other means, as the numbers are small and would likely be unreliable.

In summary, this study has demonstrated that; it is feasible to identify a high‐risk population based on estimated cumulative exposure to asbestos, that a controlled LDCT screening program is effective at identifying early‐stage lung cancer in this population, and that existing lung cancer risk prediction models do not capture this population adequately. These models need to be modified to account for occupational exposures.

## AUTHOR CONTRIBUTION

**Fraser J. H. Brims:** Conceptualization (lead); formal analysis (lead); funding acquisition (lead); methodology (lead); project administration (lead); writing – original draft (lead); writing – review and editing (lead). **Edward J. A. Harris:** Data curation (lead); validation (equal); writing – original draft (equal); writing – review and editing (equal). **Conor Murray:** Conceptualization (equal); data curation (equal); formal analysis (supporting); writing – original draft (supporting); writing – review and editing (equal). **Chellan Kumarasamy:** Data curation (supporting); formal analysis (equal); writing – review and editing (supporting). **Alice Ho:** Data curation (supporting); formal analysis (supporting); writing – review and editing (equal). **Brendan Adler:** Conceptualization (supporting); data curation (equal); formal analysis (supporting); writing – review and editing (equal). **Peter Franklin:** Conceptualization (equal); formal analysis (equal); methodology (equal); project administration (equal); validation (lead); writing – original draft (equal); writing – review and editing (equal). **Nick H. de Klerk:** Formal analysis (equal); methodology (equal); validation (equal); writing – review and editing (equal).

## CONFLICT OF INTEREST STATEMENT

None declared.

## HUMAN ETHICS APPROVAL DECLARATION

Research ethics committee approval was obtained from the University of Western Australia Health Research Ethics Committee reference RA/4/1/2119. All participants provided written informed consent. Consumer representation influenced the concept and planning for this project as part of the National Centre for Asbestos Related Diseases consumer engagement process.

Clinical trial registration: ACTRN12621001627842 at https://www.anzctr.org.au/

## Supporting information


**Appendix S1.** Methods
**Table S1.** Summary of variables utilized in the calculation of lung cancer risk through the PLCO (*n* = 933) and LLP (*n* = 1500) risk models from 1743 total participants.

## Data Availability

Deidentified data that support the findings of this study may available on request from the corresponding author. The data are not publicly available due to privacy or ethical restrictions.
