# Supplementary material for: Lung cancer screening an asbestos exposed population: Existing lung cancer risk criteria are not sufficient
Source: Respirology. 2023 Mar 8;28(6):543–50. doi: 10.1111/resp.14487 (PMC10947558; doi:10.1111/resp.14487)
Supplement: Supplementary file 1 — Appendix S1. Methods Table S1. Summary of variables utilized in the calculation of lung cancer risk through the PLCO (n = 933) and LLP (n = 1500) risk models from 1743 total participants. [file RESP-28-543-s001.docx]

**SUPPORTING INFORMATION**

**Lung Cancer Screening an Asbestos Exposed Population: Existing Lung cancer Risk Criteria are not Sufficient**

Fraser JH Brims^1,2,3^, Edward JA Harris^1,2^, Conor Murray^4^, Chellan Kumarasamary^2^ Alice Ho^1^, Brendan Adler^5^, Peter Franklin^6^ Nick H de Klerk^6^

1. Department of Respiratory Medicine, Sir Charles Gairdner Hospital, Perth, Western Australia, Australia
2. Curtin Medical School, Curtin University, Perth, Western Australia, Australia
3. National Centre for Asbestos Related Diseases, Institute for Respiratory Health, Perth, Western Australia, Australia
4. ChestRad Medical Imaging, Perth, Western Australia, Australia
5. Envision Medical Imaging, Wembley, Perth, Western Australia, Australia
6. School of Global and Population Health, University of Western Australia, Perth, Western Australia, Australia

**Appendix S1- METHODS**

**Lung function**

Post bronchodilator forced expiratory volume in 1 second (FEV_1_), forced vital capacity (FVC) and diffusion of carbon monoxide (DLCO: gas transfer) were performed in accordance with the ATS/ERS recommendations and Global Lung Initiative reference values^19, 20^

**Quantitative asbestos exposure estimates**

*Mixed occupational exposure*: we utilised a validated Australian-specific asbestos job-exposure matrix (AsbJEM), described in detail previously^23^ to estimate the cumulative asbestos exposure for the mixed cohort.

*Wittenoom exposure*: periodic measurements of airborne dust concentrations were taken from the mine, mill and township and cumulative exposure was calculated, as described previously^24^.

**Protocol for LDCT screening scans**

All CT scans were performed prone at 1.5mm slice thickness using a Siemens SOMATOM Definition FORCE machine (Siemens Healthcare, Munich, Germany), including spectral shaping of the X-ray beam with a tin filter (facilitates a ~20-30% reduction in radiation dose due to its effect eliminating radiation energies at the extremes of the spectrum which do not contribute to image quality), ultra-sensitive exotic compound detector material (Stellar Detector) and level 3 generation de-noising interactive reconstruction software (‘ADMIRE’). The scan factors were a patient size-dependent tube voltage of 100–120 kVp and tube current-time product of 20–40 mAs. Estimated radiation exposure was measured using the dose-length product (DLP) and used a conversion ‘k’ factor of 0·014 to estimate millisieverts (mSv)^23^.

All LDCT scans were routinely reported by one of three specialist thoracic radiologists blinded to exposure history, using a standardized, semi-quantitative synoptic report which was adapted from the Kusaka International Classification of HRCT for Occupational and Environmental Respiratory Diseases (ICOERD: analogous to the International Labour Organisation classification of respiratory disease)^24^. This system provides a score depending on the features or severity for different abnormalities, for instance presence of nodules, pleural plaque, emphysema, linear opacities (which represent fibrosis) and honeycombing.

The ICOERD system divides the lung fields into three zones (upper, mid, lower) and severity of emphysema and fibrosis is classified within each zone, on a scale of zero (none) to with a maximum score of 3 (marked abnormality), giving a maximum score of 18 for both lungs. The magnitude of ILA is assessed by the parameter, “linear opacity score”, with a score ≥1 considered positive for the presence of ILA (or possible fibrosis)^25^. Similarly, an “emphysema sum score” ≥1 was considered diagnostic of emphysema; subjects with normal parenchyma scores were regarded as a comparator group.

**RESULTS**

Duration of follow up prior to LDCT screening was as follows:

- Wittenoom workers:

Total follow up: 5521 years for 357 individuals

Avg follow up per person: 15.47 years.

- Wittenoom ex residents:

Total follow up: 4667 years for 227 individuals

Avg follow up per person: 20.56 years.

- Mixed occupational exposures:

Total follow up: 8032 years for 1154 individuals

Avg follow up per person: 6.96 years.

CXR was performed in the year prior to LDCT in 939 (53.9%) of the cohort with 13 lung cancers in the 12 months prior to adoption of LDCT.

Malignant mesothelioma was detected in seven participants, two had lymphoma, one metastatic prostate cancer, one melanoma with solitary pulmonary metastasis, one papillary thyroid carcinoma and one early-stage colorectal cancer (incidentally detected on FDG-PET as part of pre-operative work up for a LDCT detected lung cancer).

Table S1 below describes the variables included in the calculation of the PLCO and LLP scores.

**Table S1.** Summary of Variables Utilised in the Calculation of Lung Cancer Risk through the PLCO (n=933) and LLP (n=1500) Risk Models from 1743 total participants.

| **Variable** | **Value** | **Missing variables (n)** |
| --- | --- | --- |
| **Age** (Median, IQR) | 69.77 (63.04 – 75.73) | 0 |
| **Smoking Duration** (years)  (Median, IQR) | 21 (12 – 33) | 0 |
| **Personal Cancer History** (n) | 176 | 24 |
| **Family History of Lung Cancer** (n) | 15 | 65 |
| **COPD** (n) | 625 | 0 |
| **COPD/Pneumonia/**  **Bronchitis/Tuberculosis/**  **Emphysema** (n) | 856 | 0 |
| **Smoking Status**  (Never/Former/Current) | 596/1026/119 | 0 |
| **Education*** | 1 – 452  2 – 87  3 – 651  4 – 194  5 – 85  6 – 70 | 204 |
| **BMI** (Median, IQR) | 28.38 (25.86 – 31.45) | 35 |
| **Smoking Intensity** (Pack-Years) (Median, IQR) | 6.40 (0 – 25) | 3 |
| **Time Since Quit Smoking** (years) (Median, IQR) | 27 (12 – 37) | 0 |
| **Asbestos Exposure** (n) | 1743 | 0 |
| *Education: 1 = Less than high school graduate; 2 = High school graduate; 3 = Post high school training; 4 = Some tertiary; 5 = Tertiary graduate; 6 = Postgraduate/professional | | |
